# Mr. Henry Thomas Buckle in a New Role

**Published:** 1874-04-15

**Authors:** 


### MR. HENRY THOMAS BUCKLE IN A NEW ROLE.

MESSRS. EDITORS.—The author of "The History of Civilization in England," died after the completion of two volumes, which hardly sufficed for his introduction. He died in early manhood—a marvel of mental vigor in the esteem of his friends, and an object of envy in the eyes of his adversaries. In the language of the poet, "His mourners were two hosts—his friends and foes."

But the English speaking race, the history of whose civilization Mr. Buckle undertook to write, will hardly fail to view his character in a very different light, if due weight be given to the facts which we propose to set forth. We believe that they are incontrovertible and conclusive. Briefly, we accuse Mr. Buckle of plagiarism, and our proofs are subjoined.

In the "Transactions of the Twenty-second Anniversary Meeting of the Illinois State Medical Society, held at Rock Island, May 21st and 22d, 1872," a paper appears which bears the title, "On Organic Reform, or the Influence of Physical Organization on the Mind and Character of Man; an Address before the People and Illinois State Medical Society, at Rock Island, for the Annual Session of 1872."

As this paper is the source from which we charge that Mr. Buckle drew some of those terse and vigorous sentences, instinct with thought and fertile in suggestion, upon which his readers have lingered with increasing delight, we have merely to set side by side the original and the copy, in order to establish our proof and fix the consequent guilt upon him to whom it belongs:

| ORIGINAL PARAGRAPHS FROM THE TRANSACTIONS OF THE ILLINOIS STATE MEDICAL SOCIETY: | PLAGIARIZED PARAGRAPHS FROM BUCKLE'S HISTORY OF CIVILIZATION IN ENGLAND. |
| --- | --- |
| "Descartes, the prince of metaphysicians in his time, so far from thinking that the knowledge of external nature was essential to the discovery of truth, has laid it down as a fundamental principle, that we must separate ourselves from the delusions of nature, and even reject the evidence of our senses."—p. 182. | "Descartes .... so far from thinking that a knowledge of the external world is essential to the discovery of truth, he laid it down as a fundamental principle .... that the first step is to separate ourselves from the delusions of nature, and reject the evidence presented to our senses."—Vol. 2, pp. 88–87. |

" Early in the present century Bichat maintained that the study of the organs should be subservient to the study of the tissues composing them. ----Cuvier gained a mastery over Linnæus.----. Agassiz, in the course of his ichthyological researches, was led to perceive that the arrangement of Cuvier, according to organs, did not fulfill its purpose in regard to fossil fishes, because, in the lapse of ages the characteristics of their structure were destroyed He therefore adopted the only remaining plan, and studied the tissues, which, being less complicated than the organs, are oftener found intact. The result was the very remarkable discovery that the tegumentary membrane of fishes is so intimately connected with their organization, that if the whole of a fish has perished, except this membrane, it is practicable, by noticing its characteristics, to reconstruct the animal in its most essential parts. Of the value of this law of relation, in this connection, some idea may be formed from the fact that upon it Agassiz has based the whole of that celebrated classification of which he is the sole author, and by which fossil ichthyology has, for the first time, assumed a precise and definite shape.

" Another discovery of still greater importance, based upon Bichat's classification, is the well-known fact that the teeth of an animal have a necessary connection with the whole organization of its frame; so that, within certain limits, we can predicate the organization by examining the tooth."—pp. 184–5.

" In 1801 Bichat..published his great work on anatomy, in which the study of the organs is made altogether subservient to the study of the tissues composing them." —Vol. 2, p. 380. " Cuvier overthrew the system of Linnæus."—p. 376.

" Agassiz, who, in the course of his ichthyological researches was led to perceive that the arrangement by Cuvier according to organs, did not fulfill its purpose in regard to fossil fishes, because, in the lapse of ages, the characteristics of their structure were destroyed. He, therefore, adopted the only other remaining plan, and studied the tissues, which, being less complex than the organs, are oftener found intact. The result was the very remarkable discovery that the tegumentary membrane of fishes is so intimately connected with their organization, that if the whole of a fish has perished, except this membrane, it is practicable, by noting its characteristics, to reconstruct the animal in its most essential parts. Of the value of this principle of harmony, some idea may be formed from the circumstance that on it Agassiz has based the whole of that celebrated classification of which he is the sole author, and by which fossil ichthyology has, for the first time, assumed a precise and definite shape.

" The other discovery, of which the application is much more extensive, was made in exactly the same way. It consists of the striking fact that the teeth of each animal have a necessary connection with the entire organization of its frame; so that, within certain limits, we can predict the organization by examining the tooth."—Vol.2, pp. 383–4.

Buckle, growing bolder after pilfering these passages with impunity, proceeds to commit an act of piracy which is admirable for its audaciousness:

ORIGINAL " TRANSACTIONS."

" There has been collected a class of facts, extending over many centuries, and including many years of observations, and presented in the clearest of all forms, to wit.: in arithmetical tables, bearing directly upon this question; and what adds to their value is the fact that they, for the most part, have been gathered and arranged by government officials having no theories to establish or disclaim. Now, the actions of men may be divided into two classes, the virtuous and vicious; these classes correlate, and when taken together constitute the sum total of human actions; and whatever increases one class of actions, correspondingly diminishes the other, so that if we prove uniformity in one, we equally prove regularity in the other."—p. 186.

" The main object of government, especially of legislation, has been to protect the innocent and punish the guilty. It was, therefore, natural that European governments should study and trace the laws under which crime occurs. This investigation has now been pursued so long and so carefully, that the amount of statistics, and the commentaries accompanying them, constitute a large body of literature, well-arranged and digested." —p. 186.

"Of all the offences committed by man, we naturally infer that murder would be the most irregular and arbitrary. It is generally the crowning act of a long career of vice, and frequently happens under circumstances and impulses of the mind, which throw little, if any, light upon its cause; and hence, if the act be investigated purely as an isolated fact, it puzzles the best of minds. Some of the most heinous of these crimes are committed apparently without cause or purpose, and still the previous character of the individual does not warrant the conclusion of insanity. And when the crime is premeditated, it requires a rare combination of favorable circumstances, for which the criminal will wait and watch his opportunity, and when the favorable moment arrives—he will hesitate; there appears to be a conflict in his bosom. On the one hand, is fear of detection, of the law, and the dread penalties of religion; and perhaps he is diverted in his deed of darkness by the still small voice coming up deep from his soul, the voice of conscience. On the other hand, there is the sup-

BUCKLE'S GARBLED VERSION.

" They are based on collections of almost innumerable facts, extending over many centuries, thrown into the clearest of all forms, the form of arithmetical tables; and, finally, they have been put together by men who, being for the most part mere government officials, had no particular theory to maintain......"—Vol. 1, p. 21.

" The actions of men are, by an easy and obvious division, separated into two classes, the virtuous and the vicious; and, as these classes are correlative, and when put together compose the total of our moral conduct, it follows that whatever increases the one will, in a relative point of view, diminish the other; so that, if we can in any period detect a uniformity and a method in the vices of a people, there must be a corresponding regularity in their virtues."—Vol. 1, p. 22.

" For, the main object of legislation being to protect the innocent against the guilty, it naturally followed that European governments, so soon as they became aware of the importance of statistics, should begin to collect evidence respecting the crimes they were expected to punish. This evidence has gone on accumulating, until it now forms of itself a large body of literature, containing, with the commentaries connected with it, an immense array of facts, so carefully compiled, and so well and clearly digested. ..."—Vol. 1, p. 23..." Of all offences, it might well be supposed that the crime of murder is one of the most arbitrary and irregular. For, when we consider that this, though generally the crowning act of a long career of vice, is often the immediate result of what seems a sudden impulse;"......

" that when premeditated, its committal, even with the least chance of impunity, requires a rare combination of favorable circumstances, for which the criminal will frequently wait; that he has thus to bide his time, and look for opportunities he cannot control; that, when the time has come, his heart may fail him; that the question, whether or not he shall commit the crime, may depend on a balance of conflicting motives, such as fear of the law, a dread of the penalties held out by religion, the prickings of his posed wrong, the revenge, jealousy, personal gain, a desperation. When we put together all these thoughts, emotions, and volitions within, and the circumstances and conditions without, there arises such a combination of influences and causes that we might reasonably despair of detecting any order or method in the result of these subtle and shifting agencies, by which murder is either caused or prevented.

"But what are the facts? It now appears that murder occurs nearly as regularly, and bears as constant a relation to known causes, as the movements of the tides, or the rotations of the seasons. M. Quetelett, who spent his life in collecting and methodizing criminal statistics of various countries, states, as the result of his laborious researches: 'In everything which concerns crime, the same numbers re-occur with a certainty which cannot be misunderstood; and that this is the case of crimes which seem quite independent of human foresight, such as murders which are committed after quarrels arising from circumstances apparently casual. Nevertheless, we know from experience that every year nearly the same number of murders occur, and that the instruments by which they are committed, are employed in the same proportion.' This was the language of confessedly the first statistician in Europe; and subsequent observations have abundantly confirmed his conclusions. And even the facts seem to warrant the belief that the uniformity of crime is more clearly marked, and more capable of prediction, more closely connected with physical causes, than disease, and destruction of our bodies."—pp. 186–7.

"Another fact, still more striking, if possible, is that the number of suicides may be calculated with almost the certainty of an eclipse. Attempts to rob or murder are, or may be, baffled by second or third parties, but suicides are the isolated acts of individuals; they are more completely the product of individual volition."—pp. 187–8.

"Nor is it the crimes of men, merely, that are marked by this uniformity own conscience, the apprehension of future remorse, the love of gain, jealousy, revenge, desperation. When we put all these things together, there arises such a complication of causes, that we might reasonably despair of detecting any order or method in the result of these subtle and shifting agencies, by which murder is either caused or prevented. But now how stands the fact? The fact is, that murder is committed with as much regularity, and bears as uniform a relation to certain known circumstances, as do the movements of the tides, and the rotations of the seasons. M. Quetelet, who has spent his life in collecting and methodizing the statistics of different countries, states, as the result of his laborious researches, that 'In everything which concerns crime, the numbers re-occur with a constancy which cannot be mistaken; and that this is the case, even with those crimes which seem quite independent of human foresight, for instance, such as murders, which are generally committed after quarrels arising from circumstances apparently casual. Nevertheless, we know from experience that every year there not only take place nearly the same number of murders, but that even the instruments by which they are committed, are employed in the same proportion.' This was the language used in 1835, by confessedly the first statistician in Europe; and every subsequent investigation has confirmed its accuracy. For later inquiries have ascertained the extraordinary fact that the reproduction of crime is more clearly marked, and more capable of being predicted, than are the physical laws connected with the disease and destruction of our bodies."—Vol. 1, pp. 24–25.

"But another circumstance remains behind, still more striking. Among public and registered crimes, there is none which seems so completely dependent on the individual as suicide. Attempts to murder, or to rob, may be, and constantly are, successfully resisted. But an attempt to commit suicide, ........ becomes, as it were, isolated, ..... more clearly the product of his own volition, etc."—Vol. 1, p. 26.

"Nor is it merely the crimes of men which are marked by this uniformof sequence. The number of marriages annually contracted in England is determined more by the price of corn, and the rate of wages, than those emotional feelings of love and attachment."

"Another curious example of the law of sequence, is the aberration of memory. The post-offices of London and Paris, for many years past, have published lists of letters, the writers of which omitted to direct them; and the proportion they bear to the whole amount in each city is nearly identical from year to year; and the variation is accounted for by social and political disturbances."—p. 188.

ity of sequence. Even the number of marriages annually contracted is determined, not by the temper and wishes of individuals, ....... marriage bears a fixed and definite relation to the price of corn; and in England, instead of having any connection with personal feeling, they are simply regulated by the average earnings of the great mass of the people."

"Thus, to give a curious instance, we are now able to prove that even aberrations of memory are marked by this general character of necessary and invariable order. The postoffices of London and Paris have lately published returns of the number of letters which the writers, through forgetfulness, omitted to direct; and, making allowance for the difference of circumstances, the returns are, year after year, copies of each other."—Vol. 1, p. 32.

It may possibly be objected, to the charges sustained above, that Mr. Buckle wrote his history, and died, before the Transactions of the State Society were recorded. But so feeble an objection scarcely deserves refutation in the day when "The Mystery of Edwin Drood" is completed by its author after his death. Surely, it cannot be more difficult for a living writer to profit by the literary labors of his posterity through the medium of the spirit-world, than for a dead author to conclude, in the same manner, his volume unfinished while he was yet alive. Buckle confesses to this prophetic vision: "Once," says he, "when I first caught sight of the *whole* field of knowledge, and seemed, however dimly, to discern its various parts, and the relations they bore to each other, I was so entranced with its surpassing beauty that the judgment was beguiled, and I deemed myself able, not only to cover the surface, but also to master the details."

Who can hesitate to believe that the English author, in these words, expressly refers to the occasion upon which his judgment was beguiled into reproducing entire pages of the Transactions of an American State Medical Society, "entranced by the surpassing beauty of its details"? This latter circumstance, it is, which induces us to believe that other authors have poached upon the same preserves, and that several metaphysicians and physicists have followed Buckle's example.

In view of the approaching Convention of the State Medical Society, we desire to urge upon their attention the propriety of taking a precaution, observed in so many French publications, that of imprinting legibly upon each copy of all future Transactions, "*All Rights Reserved.*"

CURETTE.

